# Prolonged colonisation with *Escherichia coli* O25:ST131 versus other extended-spectrum beta-lactamase-producing *E. coli* in a long-term care facility with high endemic level of rectal colonisation, the Netherlands, 2013 to 2014

**DOI:** 10.2807/1560-7917.ES.2016.21.42.30376

**Published:** 2016-10-20

**Authors:** Ilse Overdevest, Manon Haverkate, Jacobien Veenemans, Yvonne Hendriks, Carlo Verhulst, Ans Mulders, Willemijn Couprie, Martin Bootsma, James Johnson, Jan Kluytmans

**Affiliations:** 1 Laboratory for Medical Microbiology, Stichting PAMM, Veldhoven, the Netherlands; 2 Laboratory for Medical Microbiology and Infection control, Amphia Hospital, Breda, the Netherlands; 3 Laboratory for Medical Microbiology and Immunology, St. Elisabeth Hospital, Tilburg, the Netherlands; 4 Julius Center for Health Sciences and Primary Care, University Medical Center Utrecht, Utrecht, the Netherlands; 5 Thebe long-term care facilities, Breda, the Netherlands; 6 Department of Mathematics, Faculty of Science, Utrecht University, Utrecht, the Netherlands; 7 Veterans Affairs Medical Centre and University of Minnesota, Minneapolis, Minnesota, USA

**Keywords:** ESBL, ST131 *E. coli*, colonization, Long-term care facility, transmission, epidemiology, *Escherichia coli*, outbreak

## Abstract

The extended-spectrum beta-lactamase (ESBL)-producing *Escherichia coli* clone ST131 (ESBL-ST131) has spread in healthcare settings worldwide. The reasons for its successful spread are unknown, but might include more effective transmission and/or longer persistence. We evaluated the colonisation dynamics of ESBL-producing *E. coli* (ESBL-EC), including ESBL-ST131, in a long-term care facility (LTCF) with an unusually high prevalence of rectal ESBL-EC colonisation. During a 14-month period, rectal or faecal samples were obtained from 296 residents during six repetitive prevalence surveys, using ESBL-selective culture. Transmission rates, reproduction numbers, and durations of colonisation were compared for ESBL-ST131 vs other ESBL-EC. Furthermore, the likely time required for ESBL-ST131 to disappear from the LTCF was estimated. Over time, the endemic level of ESBL-ST131 remained elevated whereas other ESBL-EC returned to low-level prevalence, despite comparable transmission rates. Survival analysis showed a half-life of 13 months for ESBL-ST131 carriage, vs two to three months for other ESBL-EC (p < 0.001). Per-admission reproduction numbers were 0.66 for ESBL-ST131 vs 0.56 for other ESBL-EC, predicting a mean time of three to four years for ESBL-ST131 to disappear from the LTCF under current conditions. Transmission rates were comparable for ESBL-ST131 vs other ESBL-EC. Prolonged rectal carriage explained the persistence of ESBL-ST131 in the LTCF.

## Introduction

The prevalence of extended-spectrum beta-lactamase (ESBL)-producing *Enterobacteriaceae* is increasing rapidly worldwide [1,2]. Infections with these and other resistant bacteria are associated with higher morbidity, mortality, and healthcare costs [3,4]. *Enterobacteriaceae* colonising the gut are the most important reservoirs for infection and transmission of ESBL-producing *Enterobacteriaceae* [5,6].

The first reports of outbreaks with ESBL-producing *Enterobacteriaceae* came from hospitals. However, more and more outbreaks are reported in long–term care facilities (LTCFs) [7,8]. Residents of LTCFs are mainly frail, elderly people, with underlying diseases who often have medical devices and need regular medical care. Among these residents, a low functional status and higher medical and nursing dependence are associated with a greater risk of ESBL carriage [9]. For their residents, LTCFs emphasise the quality of life, including participation in social activities, over healthcare. Therefore, the amount of interaction between LTCF residents is higher than between hospitalised patients. This may be an important factor for transmission since the risk of transmission of ESBL-producing *Enterobacteriaceae* is greater among household contacts than among hospital inpatients [10]. Furthermore, our own experience shows that diagnostic sampling frequency in LTCFs is low and infection control measures are not as strict as in hospitals. We assume that, under these conditions, transmission of ESBL-producing organisms between residents could often be overlooked.

From June to July 2012, a routine prevalence survey involving nine LTCFs in the southern Netherlands identified one facility with an unusually high prevalence of rectal ESBL carriage of 21%, compared with 0–10% for six of the other LTCFs [11] and ca 5% for hospitalised patients [12]. The two remaining LTCFs of the 2012 prevalence survey housed a small number of residents and had a prevalence of ESBL carriage of 14 and 18%. In the high ESBL-prevalence LTCF, strain typing showed the presence of one large cluster of ESBL-producing *Escherichia coli* from sequence type O25:ST131 (i.e. ESBL-ST131), along with other smaller clusters and unique strains. In accordance with Dutch guidelines [13], this prompted outbreak containment measures, including frequent prevalence surveys.

The ESBL-ST131 clonal lineage is a major driver of the current worldwide spread of ESBLs [14-16]. It is associated with presence of multiple virulence factors [17] and with community-acquired infections. Older age and LTCF residence have been implicated as independent risk factors for ESBL-ST131 colonisation and infection [18]. ST131 was the most prevalent clone in a recent study of antimicrobial resistance in another Dutch LTCF [19].

Here we evaluated, over a period of 14 months, the epidemiology of various ESBL-producing *E. coli* (ESBL-EC), including ESBL-ST131, in a LTCF with a high endemic level of ESBL carriage. Specifically, we assessed whether ESBL-ST131 strains were more transmissible or more persistent colonisers than other ESBL-EC. Both factors are theoretical explanations for the successful worldwide spread of ESBL-ST131.

## Methods

### Study period and setting

We evaluated the dynamics of colonisation with ESBL-producing *Enterobacteriaceae* among residents of a LTCF in the Netherlands over 14 months, from March 2013 to April 2014. The LTCF comprised four semi-separate buildings (A, B, C, and D), each divided into one to three separate wards (A1–3, B1–2, C1–3, and D). Each ward housed ca 20 residents and contained two kitchens and communal areas. Sanitary facilities were shared by several residents each. Nursing staff was dedicated to specific wards. The building contained communal recreation and therapy areas where residents from all buildings and wards met regularly.

During the study period, improved infection control measures, improved emphasis on hand hygiene, and improved cleaning strategies were implemented in all wards, irrespective of the prevalence or clonal distribution of ESBL colonisation. No attempts were made to actively decolonise residents.

### Specimen collection

Over the 14 month study period, six cross-sectional surveys were performed at intervals of two to three months by culturing faeces or rectal swabs from all residents. For residents admitted during the study, efforts were made to culture them similarly within one week after admission.

In order to assess possible routes of transmission, the following cultures were obtained concurrently with the faecal surveys in residents: environmental cultures (5 times), hand cultures (twice from all available staff, once from residents), and air sedimentation cultures (twice near ESBL-colonised residents and a selection of non-colonised residents).

### Identification and detection of resistant strains

Faecal and rectal samples were collected using ESwab (Copan diagnostics, Brescia, Italy). For environmental cultures, standardised surfaces of 10x10 cm were sampled thoroughly using ESwab medium in the first two surveys, and a sterile 10x10 cm pad soaked in sterile isotonic saline solution for the subsequent three surveys. Hands of staff members were cultured by having the workers dip and rub their hands into tryptic soy broth (TSB). Residents’ hands were cultured using ESwab, with special attention paid to palms, fingers, nails, and jewellery. Air sedimentation cultures were performed by placing five selective agar plates (EbSA agar, Cepheid Benelux, Ledeberg, Belgium) in close proximity to residents while they were being washed and getting dressed.

The sterile pads for environmental cultures and all ESwabs were incubated for 16 to 18 hours at 37 °C in 15 mL of TSB containing 8 mg/L vancomycin and 0.25 mg/L cefotaxime. Then, 10 µL of the broth was inoculated and incubated on an EbSA agar plate (Cepheid Benelux, Ledeberg, Belgium), selective for ESBL-producing *Enterobacteriaceae*. The TSB used to rinse staff members’ hands was incubated directly, as were the agar plates used for air sedimentation culture.

Identification of all oxidase-negative, Gram-negative bacteria was performed by matrix-assisted laser desorption/ionization–time (MALDI-TOF, BioMérieux, Marcy l’Etoile, France). Susceptibility testing was performed by VITEK2 (BioMérieux, Marcy l’Etoile, France) using the European Committee on Antimicrobial Susceptibility Testing (EUCAST) criteria [20], and ESBL production was confirmed by a double disk method [21].

### Typing

All phenotypically confirmed ESBL-EC underwent phylogroup-defining PCR [22]. Group B2 *E. coli* underwent O25:ST131-specific PCR [23].

ESBL-EC obtained from colonisation cultures, environmental cultures, hand cultures and air sedimentation cultures underwent ESBL genotyping using a micro-array (Check-MDR CT103, CheckPoints, Wageningen, the Netherlands) [24,25] and strain typing by using amplified fragment length polymorphism (AFLP) [26]. Clusters were defined based on both visual and computerised interpretation of AFLP patterns.

Of residents with repetitive positive colonisation cultures with similar ESBL-EC, only the first isolate was genotyped. Similarity was defined as identical species, identical phylogroup and O25:ST131 status, and absence of major differences in susceptibility (i.e. susceptible vs resistant) for the 25 antibiotics tested.

### Statistical analysis

Acquisition was defined as detection of an ESBL-producing organism in a previously culture-negative resident. Transmission was defined as acquisition of an ESBL-EC strain identical according to AFLP profile and ESBL-variant to one already present on the ward where the individual resided before the acquisition. Routine prevalence surveys in several LTCFs [11] and a hospital (data not shown) in the same area as the LTCF studied showed little clustering of ESBL-EC and low prevalence of colonisation with O25:ST131 *E. coli*. Consequently, it is unlikely for newly admitted residents to be colonised with the same strain as present on the ward they are admitted to. Transmission was thus also assumed for residents who were admitted during the study period, stayed on a ward over 14 days before being cultured, and, who were found to be colonised with an ESBL-EC strain already present on that ward when first cultured.

We used differences in length of stay (LOS) as a marker for inter-individual differences in susceptibility to colonisation. We reasoned that if differences in susceptibility were present, residents less susceptible to colonisation should remain non-colonised for a longer LOS than other residents and consequently acquisition risk should be lower for patients with a longer LOS. For the analysis, LOS was grouped into three-month periods in which residents could be ESBL-culture-negative and at risk for acquisition, or could have acquired ESBL-EC. Differences in acquisition risk between a LOS shorter vs one longer than 12 months were assessed by Chi-Square analysis.

Median duration of colonisation was calculated from the first positive culture using Kaplan-Meier survival analysis, with status ‘loss of colonisation’ as the primary outcome. Differences between ESBL-ST131 and other ESBL-EC were tested with Log-Rank analysis. Residents acquiring colonisation in the final prevalence survey were excluded.

Transmission rates and corresponding reproduction numbers were calculated for ESBL-ST131 and other ESBL-EC separately, taking into account the ward-level infection pressure and assuming that transmission occurred only at the ward. Residents were considered to have newly acquired or lost colonisation on the day of the culture that detected their changed colonisation status. Weighted days at risk were calculated by multiplying, for each day, the number of positive (i.e. colonised) residents per ward by the number of non-colonised residents on the same ward. Weighted days at risk were summed over all wards, separately for all combinations of AFLP plus ESBL-variant. Per-day transmission rates were calculated by dividing the number of presumed transmissions by weighted days at risk. A per-admission reproduction number was calculated by multiplying the number of residents on a ward (n = 20) by the per-day transmission rates of ESBL-ST131 and other ESBL-EC and the corresponding mean durations of colonisation obtained from the Kaplan-Meier survival analysis [27].

The time for ESBL-ST131 to disappear from the LTCF was estimated by using a mathematical model that incorporated the per-day transmission rate and a constant decolonisation rate equal to the mean duration of colonisation obtained from the Kaplan-Meier survival analysis. The model randomly simulated one million scenarios. This was repeated for situations with one to 10 colonised residents per ward. Additionally, the effects of alterations in the transmission rates and mean duration of colonisation on time for all ESBL-ST131 to disappear from the LTCF were calculated.

### Ethical considerations

Data for this study were obtained as part of outbreak containment. Frequent prevalence surveys are part of the measurements recommended by the Dutch guidelines [13]. No informed consent was obtained, but residents were informed about the surveys and had the option to refuse sampling.

## Results

### Colonisation cultures

During the study period, the LTCF housed a total of 296 residents, 126 male and 170 female, with an average of 173 residents at the time of the prevalence surveys. During the study period, 125 residents were newly admitted and 120 residents were lost to follow-up due to transfer to other facilities, transfer to home, or death. The average age at time of the prevalence surveys was 78 years (range: 46–98 years, SD: 11 years). The participation rate was 93.7% (964/1,029) for intended culturing at the prevalence surveys and 66.9% (83/125) at admittance. Only four residents declined to participate at all culture points.

In total, 1,050 rectal or faecal samples were obtained. Of these, 188 (17.9%) yielded one or more ESBL-EC, including 131 (12.5%) with ESBL-ST131 and 57 (5.4%) with other ESBL-EC. The 131 ESBL-EC-positive rectal samples were obtained from 69 different residents (23.3% of 296). Table 1 shows the number of residents who were colonised at the start of the survey, acquired colonisation during the study, or were already colonised when admitted during the study period.

**Table 1:** Number of residents colonised with extended-spectrum beta-lactamase-producing *Escherichia coli* at various study points in a long-term care facility, the Netherlands, March 2013 to April 2014 (n=296)

Organism category	Residents colonised at start of the survey (number positive during entire survey^a^)	Residents with colonisation acquired during study period (number with presumed in-ward transmission)	Residents colonised when admitted during the study period	Total number colonised at any point
ESBL-ST131^b^	24 (10)	14 (12)	3	41
Other ESBL-EC^c^	11 (1)	17 (10)	5	33
Total	35 (11)	29^d^ (22)	8	69^d,e^

EC: *Escherichia coli*; ESBL: extended-spectrum beta-lactamase.

^a^ Only residents who remained in the facility for the entire study period could be positive during the entire survey; this in contrast to residents who were lost to follow-up.

^b^ ESBL-ST131: ESBL-producing *E. coli* isolates belonging to sequence type ST131.

^c^ Other ESBL-EC: ESBL-producing *E. coli* isolates not belonging to ST131.

^d^ Two residents acquired both an ESBL-ST131 and a non-ST131 ESBL-EC.

^e^ Three residents were positive with ESBL-ST131 at the start of the survey, and acquired a non-ST131 ESBL-EC later.

All ESBL-ST131 isolates (100%; 131 isolates from 69 residents) were resistant to ciprofloxacin vs 25 of 57 (44%; p<0.001) other ESBL-EC isolates obtained from 15 of 32 (47%; p<0.001) residents colonised with other ESBL-EC. In contrast, only 19 of 131 (15%) ESBL-ST131 isolates, obtained from nine of 69 residents (13%), were resistant to co-trimoxazole vs 43 of 57 (75%; p<0.001) other ESBL-EC isolates, obtained from 26 of 32 (81%; p<0.001) colonised residents. No resistance to colistin, meropenem or imipenem was observed in any of the isolates.

The prevalence of ESBL-EC colonisation was unevenly distributed across the LTCF. At study onset, wards B-1, B-2, and C-2 had large clusters with ESBL-ST131 (29 carriers, all with isolates from the same AFLP cluster; ward prevalence 39–45%). Wards A-2, A-3, and C-3 had smaller clusters of other ESBL-EC plus sporadic carriage of non-related isolates (16 carriers; ward prevalence 11–23%). The remaining three wards had only sporadic cases of ESBL-EC colonisation (2 carriers; ward prevalence < 5%). During the follow-up period, the endemic level of ESBL-ST131 remained high, and on ward A-3 new colonisation and transmission of ESBL-ST131 appeared. However, in the same period the prevalence of other ESBL-EC decreased with some sporadic cases remaining. On wards C-1 and D, the prevalence of ESBL colonisation remained at zero.

### Environmental surveys

Of 485 standardised environmental cultures, 17 (3.5%) yielded ESBL-EC, including 17 (6.0%) of 285 done in the last three of five surveys using the sterile gauze method, vs none (0%) done in the first two surveys using the Eswab method (p < 0.001). Isolates from only nine of the 17 positive cultures matched isolates obtained from residents on the same ward during the same survey. Three identical environmental ESBL-EC isolates were obtained from ward D, but in that time period, no prevalence survey was performed on this ward. Toilets were the sites most likely to yield any ESBL-EC (11 of 17 positive sites), and overall ESBL-ST131 was less often cultured than were other ESBL-EC (4 vs 13 of 285 cultures; Table 2).

**Table 2:** Prevalence of surface contamination with extended-spectrum beta-lactamase-producing *Escherichia coli*, long-term care facility, the Netherlands, March 2013 to April 2014

Surface	Total number of cultures^a^	Number of cultures positive^a^ (row %)		
		Total	ESBL-ST131	Other ESBL-EC
Toilet or bedside commode	103	11 (10.7)	3 (2.9)	8 (7.8)
Sink	54	2 (3.7)	1 (1.9)	1 (1.9)
Kitchen area	48	3 (6.3)	0	3 (6.3)
Common living area	58	1 (1.7)	0	1 (1.7)
Total	285	17 (6.0)	4 (1.4)	13 (4.6)
Ward-related^b^	285	9 (3.2)	4 (1.4)	5 (1.8)

ESBL-ST131: ESBL-producing *E. coli* isolates belonging to sequence type ST131; Other ESBL-EC: ESBL-producing *E. coli* isolates not belonging to ST131.

^a^ Only the results from the sterile gauze method are shown.

^b^ Number of isolates that by genomic profiling were similar to isolates obtained from residents’ colonisation cultures during the same time-period.

### Hand and air sedimentation cultures

Hand cultures from four (2.7%) of 148 cultured staff members yielded ESBL-producing *Enterobacteriaceae*. All four individuals worked on ward C-2, a ward with a high endemic level of ESBL-ST131. However, only one of them carried ESBL-ST131 on the hands; the other three all carried *bla*_CTX_M9_-producing *Enterobacter cloacae,* another strain present in a colonised resident on ward C-2.

Of 176 residents, 168 (95.5%) underwent hand culturing. At the time of hand culture, 30 (17.9%) of these residents were colonised with ESBL-EC, and three (1.8%) had unknown colonisation status. Hand cultures of only two residents yielded an ESBL-producing organism, in each instance, non-*E. coli*. For only one of these residents the cultured strain, a *bla*_CTX-M9_-producing *Enterobacter cloacae*, corresponded with a strain found in rectal colonisation cultures obtained from two other ward residents.

Air sedimentation cultures were obtained near 52 residents, including all 26 ESBL carriers plus 26 non-colonised residents. In the vicinity of three of these residents, air sedimentation cultures were positive for the ESBL-ST131 strain they were colonised with. Repeated air sedimentation cultures for these three residents, and for 12 other ESBL-carriers, were negative.

### Length of stay as marker for inter-individual differences in acquiring extended-spectrum beta-lactamase-producing *Escherichia coli* colonisation

**Figure 1:**
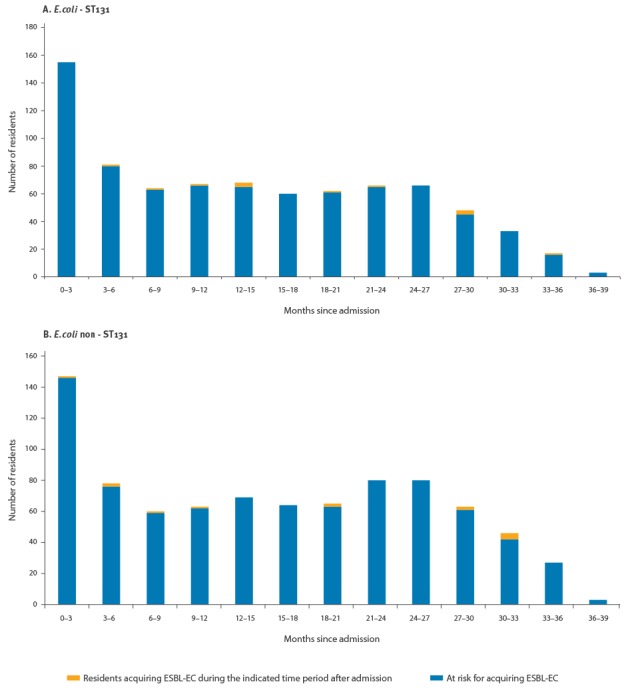
Acquisition of extended-spectrum beta-lactamase-producing *Escherichia coli* colonisation at various lengths of stay, long-term care facility, the Netherlands, March 2013 to April 2014

The risk of acquiring ESBL-EC did not vary in relation to LOS; prolonged LOS did not select for residents less susceptible to acquiring ESBL-colonisation (Figure 1). For both ESBL-ST131 and other ESBL-EC, acquisition risk did not differ between residents with a LOS shorter vs longer than 12 months (p = 0.13 and p = 0.84, respectively).

ESBL-ST131: ESBL-producing *E. coli* isolates belonging to sequence type ST131; Other ESBL-EC: ESBL-producing *E. coli* isolates not belonging to ST131.

The histogram shows the number of residents during the study period with a length of stay equal to or greater than the indicated number of months. Residents already admitted at the start of our survey ‘entered’ the histogram at the time of their first negative culture.

### Duration of colonisation

**Figure 2:**
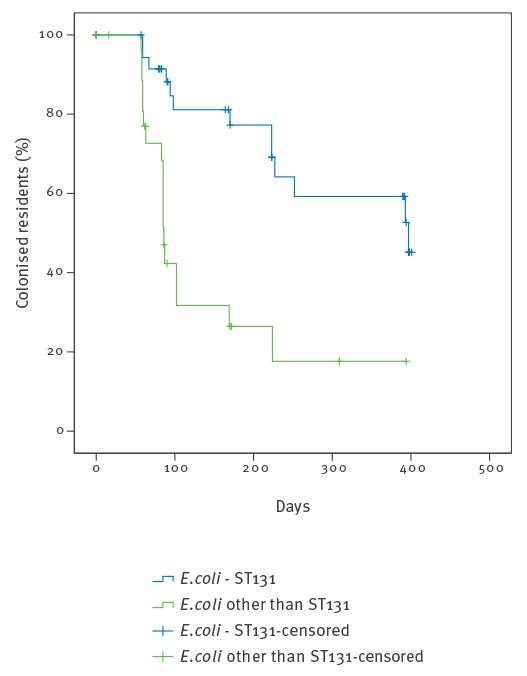
Survival curve for remaining colonised with extended-spectrum beta-lactamase-producing *Escherichia coli*, long-term care facility, the Netherlands, March 2013 to April 2014

During the study, conversion to ESBL-negative was observed for 13 of 39 ESBL-ST131 carriers, vs 18 of 29 carriers of other ESBL-EC (p = 0.03). Survival analysis showed that the half-life of carriage for ESBL-ST131 was 13 months, compared with two to three months for other strains (p < 0.001; Figure 2).

ESBL: extended-spectrum beta-lactamase.

Day 0 is the day of a resident's first rectal culture with ESBL-producing *Escherichia coli*. Follow-up ends on the day of the first negative rectal culture (drop of the line) or at the day the resident dies or leaves the facility ( + sign on the curve).

### Transmission rates

During the study, we documented 12 transmissions involving ESBL-ST131 and 10 involving other ESBL-EC. The ratio of per-day transmission rates for ESBL-ST131 vs other ESBL-EC was 0.59 (95% (CI): 0.26–1.32), indicating that other ESBL-EC spread as fast as or more readily compared with ESBL-ST131. The corresponding reproduction numbers were 0.66 (95% CI: 0.25–1.09) for ESBL-ST131 and 0.56 (95% CI: 0.20–1.01) for other ESBL-EC.

### Estimated duration for extended-spectrum beta-lactamase-producing *Escherichia coli*-ST131 to disappear from the long-term care facility

**Figure 3:**
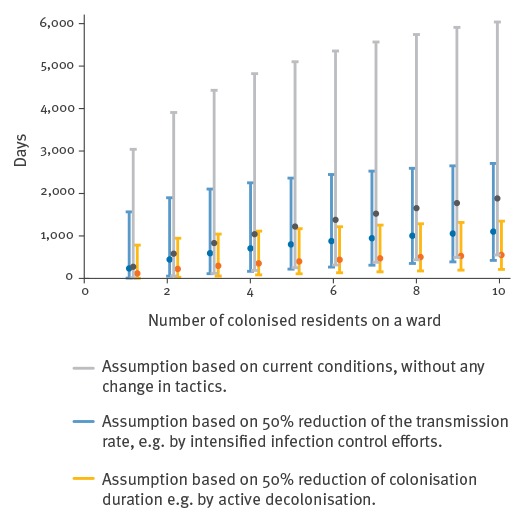
Estimated time for all extended-spectrum beta-lactamase-producing *Escherichia coli*-ST131 to disappear from a ward and effect of transmission rate and duration of colonisation, long-term care facility, the Netherlands, March 2013 to April 2014

Figure 3 shows the estimated time for ESBL-ST131 to disappear from a ward, based on the number of colonised residents, the mean duration of colonisation, and the estimated reproduction numbers. In the situation observed during the study, with a maximum of six colonised residents per ward, the mean expected time for all ESBL-ST131 to disappear from the LTCF would be more than 1,000 days, or three to four years. Halving the duration of colonisation, e.g. by active decolonisation, would reduce the average expected time to ca 400 days (1 year), whereas halving the transmission rate, e.g. by improved hygiene, would reduce it only to 800 days (2 to 3 years).

ESBL: extended-spectrum beta-lactamase

Dots: mean time for all ESBL-ST131 to disappear from a ward.

Vertical lines: 95% confidence intervals.

## Discussion

We performed a prospective cohort study of ESBL colonisation, comparing ESBL-ST131 with other ESBL-EC, in a LTCF. In the studied LTCF a high endemic level of ESBL-ST131 colonisation persisted in spite of measures taken, while colonisation with other ESBL-EC returned to a more normal level over time. We documented prolonged colonisation of individual residents with ESBL-ST131, with a half-life of ca 13 months compared to two to three months for other ESBL-EC (p < 0.001). This appeared to sustain the high endemic level of ESBL-ST131 colonisation.

As alternative explanations for the persistence of ESBL-ST131, we examined the environment, hands of staff members, and direct resident-to-resident contact as possible transmission routes. Strikingly, we found that ESBL-ST131 were nearly absent from the corresponding cultures, whereas other ESBL-EC were more often detected; thus environmental contamination with ESBL-ST131 did not explain the sustained ESBL-ST131 colonisation. Furthermore, transmission rates did not differ between ESBL-ST131 and other ESBL-EC, which excluded another possible explanation for the findings. Prolonged gut colonisation appeared to be the sole explanation for the sustained high prevalence of ESBL-ST131.

The reasons for prolonged ESBL-ST131 colonisation are unclear but can be speculated upon. ESBL-ST131 may have an intrinsically better ability to persist in the gut than other ESBL-EC, and this may be even more pronounced in elderly or functionally dependent individuals who constitute most of the population in LTCFs. Further research should be performed to elucidate the mechanisms underlying prolonged colonisation by ESBL-ST131.

Since the study data were collected in the context of infection prevention-related interventions, data on patient-specific factors such as functional status or indwelling catheters could not be obtained. We used difference in acquisition risk for different LOS as a surrogate marker for differences in acquisition risk between residents. However, longer LOS did not select for residents less susceptible to acquisition of ESBL-EC, indicating that differences in susceptibility between patients with ESBL-ST131 vs other ESBL-EC were unlikely.

The per-admission reproduction numbers for ESBL-ST131 and other ESBL-EC were comparable at 0.66 (95% CI: 0.25–1.09) and 0.56 (95% CI: 0.20–1.01), respectively. Both were less than one, indicating that, with the infection control measures in place, the prevalence of ESBL carriage should eventually return to baseline. In the situation where residents have a long average LOS and an endemic strain (ESBL-ST131) is present that that causes persistent colonisation, and infection control measures are in place, the estimated duration for ESBL-ST131 to disappear from the LTCF is three to four years. This indicates that prolonged periods of increased prevalence do not necessarily mean that infection control measures are ineffective.

Statistical modelling predicted that the time required for ESBL-ST131 to disappear from the LTCF would be affected only minimally by improved infection control measures. In contrast, a shortened duration of colonisation e.g. by decolonisation would, have a larger impact. Unfortunately, reliably effective decolonisation strategies for ESBL carriage are unavailable. Probiotics [28] and donor faeces infusion [29] have been used in experimental settings, and selective bowel decontamination (SDD) regimes have been proposed [30,31]. However, some studies observed only a temporary suppression of ESBL carriage during SDD treatment, with a rapid rebound one week after the end of treatment [32].

Few reports of prolonged colonisation with ESBL-EC have been published. Alsterlund et al. reported five residents who remained colonised for 41 to 59 months after an infection caused by ESBL-EC [33]. Other authors reported colonisation durations of 1.4 months [34], more than three months [35], and of 179 days (i.e. ca  6 months) [36]. Prolonged carriage of ESBL-producing bacteria after travel has also been documented [37]. To our knowledge, only one study compared duration of colonisation for different types of ESBL-producing *Enterobacteriaceae*. Titelman et al. found that faecal carriage of ESBL-EC persisted in 26 of 61 patients one year after infection, and that prolonged carriage is associated with *E. coli* phylogroup B2 [38]. In our study, the prolonged duration of colonisation was ascribed solely to ESBL-ST131 (phylogroup B2), with a 13-month colonisation half-life, vs three to four months for other phylogroup B2 *E. coli*.

Differences in transmission rates between different types of ESBL-EC have been investigated previously. Hilty et al. suggested that *E. coli* phylogroups B2 and D are more often transmitted within households than phylogroups A and B1 [10]. However, these differences were not statistically significant (p = 0.10) ), and clonal typing (e.g. to identify ST131) was not done. Adler et al. found that CTX-M-27 (CTX-M-9 group)-producing ST131 *E. coli* spread more efficiently than the CTX-M-15 ST131 *E. coli* [39]. Since our cohort included only few CTX-M-9 group-positive ST131 isolates, we could not reliably compare these two ST131 subgroups.

Our analysis has several limitations. Firstly, we assume that all residents are equally contagious over time, whereas, hypothetically, superspreading events or periods of increased infectiousness may occur. Secondly, we used a conservative definition for ‘transmission’ that presumed that transmission occurred only between residents on the same ward and disregarded the possibility of plasmid transmission. The resulting transmission numbers, which might have been underestimates, were used to calculate reproduction numbers, which if too low could have resulted in underestimation of the average duration for ESBL-ST131 to disappear from the LTCF. Thirdly, the possibility of new introductions of ESBL-ST131, for example through food, was not taken into account, which could also have resulted in an underestimation of the average duration of ESBL-ST131 to disappear from the LTCF. On the other hand, the method used to type the isolates (AFLP) is not as specific as, for example, whole genome sequencing (WGS). Theoretically, this might have led to an overestimation of transmissibility by assigning isolates to the same clonal complex that actually represented different clonal lineages. However, when used in prevalence surveys in other healthcare facilities in the same area and time period, AFLP revealed hardly any clonal relatedness. Therefore, the clonal relatedness in this specific LTCF is likely to represent clonal spread.

Another limitation is the setting, i.e. a specific LTCF during a period of elevated endemic levels of ESBL colonisation that triggered intensified infection control measures. Transmission rates and duration of colonisation might be different in other situations. However, we suspect that the observed differences in colonisation duration between ESBL-ST131 and other ESBL-EC can be extrapolated reasonably to other settings. Lastly, we had no data on underlying disease or use of medical devices or antimicrobials during the study period. From a previous study we know that in this LTCF use of antimicrobial and medical devices is infrequent [11]. Therefore we think that these factors cannot explain why ESBL-ST131 has caused such a high endemic level of colonisation in the LTCF.

Our study also had notable strengths. Most important is the length of follow-up (14 months), with clearly defined intervals at which standardised cultures were taken, and the high participation rate (90.6%).

In conclusion, we found that ESBL-ST131 can colonise LTCF residents for prolonged periods, with an estimated half-life of 13 months, which contrasts with the two to three month half-life of other ESBL-EC. Furthermore, calculated transmission rates did not differ between ESBL-ST131 and other ESBL-EC, and environmental contamination was actually more abundant for other ESBL-EC than for ESBL-ST131. Therefore, duration of colonisation was the main identified factor that contributed to the success of ESBL-ST131 in this LTCF under the current infection control measures. We postulate that prolonged colonisation may also be the key to success of this clone worldwide, which merits further study. Our models predict that implementing additional infection control measures aimed at limiting the spread of ESBL-ST131 would have only a minor effect on high colonisation prevalence levels, whereas effective decolonisation strategies should have a much more profound effect. Therefore, in addition to implementing infection control measures, development of effective decolonisation strategies is warranted to contain the spread of ESBL-ST131 worldwide.
